# Reporting of loss to follow-up information in randomised controlled trials with time-to-event outcomes: a literature survey

**DOI:** 10.1186/1471-2288-11-130

**Published:** 2011-09-21

**Authors:** Elke Vervölgyi, Mandy Kromp, Guido Skipka, Ralf Bender, Thomas Kaiser

**Affiliations:** 1 Institute for Quality and Efficiency in Health Care (IQWiG), Dillenburger Str. 27, 51105 Cologne, Germany; 2 Faculty of Medicine, University of Cologne, Joseph-Stelzmann-Str. 20, 50931 Cologne, Germany

**Keywords:** randomised controlled trial, survival analysis, Kaplan-Meier plot, loss to follow-up

## Abstract

**Background:**

To assess the reporting of loss to follow-up (LTFU) information in articles on randomised controlled trials (RCTs) with time-to-event outcomes, and to assess whether discrepancies affect the validity of study results.

**Methods:**

Literature survey of all issues of the BMJ, Lancet, JAMA, and New England Journal of Medicine published between 2003 and 2005. Eligible articles were reports of RCTs including at least one Kaplan-Meier plot. Articles were classified as "assessable" if sufficient information was available to assess LTFU. In these articles, LTFU information was derived from Kaplan-Meier plots, extracted from the text, and compared. Articles were then classified as "consistent" or "not consistent". Sensitivity analyses were performed to assess the validity of study results.

**Results:**

319 eligible articles were identified. 187 (59%) were classified as "assessable", as they included sufficient information for evaluation; 140 of 319 (44%) presented consistent LTFU information between the Kaplan-Meier plot and text. 47 of 319 (15%) were classified as "not consistent". These 47 articles were included in sensitivity analyses. When various imputation methods were used, the results of a chi^2^-test applied to the corresponding 2 × 2 table changed and hence were not robust in about half of the studies.

**Conclusions:**

Less than half of the articles on RCTs using Kaplan-Meier plots provide assessable and consistent LTFU information, thus questioning the validity of the results and conclusions of many studies presenting survival analyses. Authors should improve the presentation of both Kaplan-Meier plots and LTFU information, and reviewers of study publications and journal editors should critically appraise the validity of the information provided.

## Background

Kaplan-Meier plots are frequently used in articles on studies analysing survival (time-to-event) data. The corresponding key paper by Kaplan and Meier [1] is one of the most frequently cited statistical articles [2] (34,191 citations in ISI Web of Knowledge^®^, http://www.isiknowledge.com, 22.09.2010**)**. The Kaplan-Meier method estimates the probability of survival at a given time point for a member of the population from which the sample is drawn [3], taking into account patients who did not experience the event (outcome) of interest. These patients are classified as censored. Censoring may occur if a patient reaches the planned end of study, or is lost to follow-up [4]. A Kaplan-Meier analysis is only unbiased if the main assumptions hold that firstly, survival probabilities are the same at any given point in time both for patients who are censored and those who continue the study, and secondly, survival probabilities are the same independent of the time of recruitment [3].

Recommendations for the presentation and interpretation of survival plots are given in the literature. For example, key information on follow-up can be presented by displaying the numbers still at risk of the event in each treatment group [5]^,^[6], by giving a summary measure of follow-up (e.g. median or range of follow-up) [5]^,^[6], and by marking the times of censored observations on the survival curve in smaller studies [6]. However, despite these recommendations, previous reviews of survival analyses published in medical journals have shown substantial reporting deficits [7-9].

We also found reporting deficits in studies presenting survival analyses included in reports from our Institute [10,11], i.e. inconsistencies between loss to follow-up (LTFU) information derived from Kaplan-Meier plots and reported in the text of study publications. Large numbers of LTFU patients create the problem of increasing the variance of estimated treatment effects. Unequal LTFU proportions between groups raise doubts about the conduct of the study and hence the validity of the results.

The main objective of this survey is to assess the consistency of LTFU information derived from Kaplan-Meier plots and reported in the text of articles on randomised controlled trials (RCTs) in four leading general medical journals. We also assessed the impact of discrepancies in LTFU information on the validity of study results.

It should be noted that there is great variability concerning the definition of LTFU [12]. In the Cochrane glossary this term is defined as "the loss of participants during the course of a study" (and also called "attrition" or "dropouts") [13]. Following this definition, in the present publication we use this term for any patient who "was lost" i.e. discontinued the study prematurely for any reason.

## Methods

A sensitive search of PubMed was performed to identify RCTs published in four leading medical journals between 1 January 2003 and 31 December 2005 (BMJ, JAMA, Lancet, and the New England Journal of Medicine [NEJM]). The search was limited to citations with abstracts. The search strategy is available in Additional file 1.

All full texts of retrieved RCTs were then screened to identify eligible articles, i.e. RCTs including at least one Kaplan-Meier plot presenting a comparison of two or more therapies. One Kaplan-Meier plot from each eligible article was assessed, preferably a plot displaying the outcome "all-cause mortality" (or a composite outcome including all-cause mortality). If no mortality outcome was reported, the primary endpoint was used.

Data were extracted using an extraction form that is available from the authors on request. The items extracted were: (1) definition and number of events of interest and competing events; (2) information on numbers of patients (for each group separately, if possible) (a) randomised, (b) analysed, (c) with incomplete follow-up, and (d) at risk; (3) minimum duration of follow-up (preferably the actual duration, or if not available, either the duration estimated by means of the period between end of enrolment and end of study or the planned duration).

In articles including information on all items above, the numbers of LTFU patients in each group can be inferred from the Kaplan-Meier plot if numbers at risk are given at a time point before minimum follow-up. These articles were classified as "assessable". In some articles details on LTFU can also be inferred even if information on some items is missing. For instance, in small studies each patient can be identified in the plot. These articles were also classified as "assessable". The remaining publications were classified as "not assessable".

**Figure 1:**
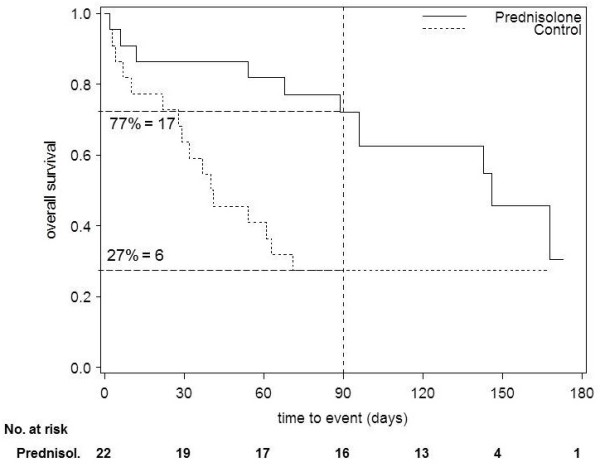
**Recalculation of the numbers at risk - example classified as "consistent"**. Kaplan-Meier plot of a randomised trial comparing prednisolone and a control group [4]. According to the information in the text of the publication, one patient was lost to follow-up in the prednisolone group and minimum follow-up was 120 days. At one time point beforehand (90 days), we read the survival probability from the curve (see vertical line). We recalculated the number of patients at risk by multiplying the survival probability with the number of randomised patients (number at risk: 17 in the prednisolone group vs. 6 in the control group). As the calculated and reported numbers matched (taking into account the one patient lost to follow-up in the prednisolone group), this example would be classified as "consistent".

**Figure 2:**
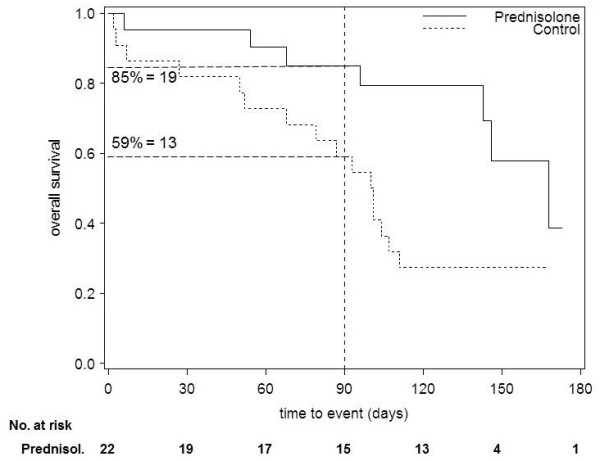
**Recalculation of the numbers at risk - example classified as "not consistent"**. Kaplan-Meier plot of the trial presented in figure 1, but using fictive data. In this example it is assumed that no patient was reported as lost to follow-up in either group and minimum follow-up was 120 days. As in figure 1, we read the survival probability from the curve at 90 days. We multiplied the survival probability by the number of randomized patients in order to recalculate the number of patients at risk (number at risk: 19 in the prednisolone group vs. 13 in the control group). As the reported number at risk was smaller in the prednisolone group, four patients must have been censored before day 90. As no losses to follow up were reported, this fictive example would be classified as "not consistent".

Assessable articles underwent further evaluation: At the last time point with information on numbers at risk before the time of minimum follow-up ("time point t"), the survival probability was read from the curve. As no patient should be censored before time point t, the Kaplan-Meier curve represents 1 minus the empirical failure distribution function. The numbers of patients who still ought to be at risk at time point t can be calculated by multiplying the survival probability with the number of randomised patients (see Figure 1 for an example calculation). If the calculated number of patients at risk was higher than the numbers at risk reported in the figure legend, we tried to solve this discrepancy by considering information on LTFU reported in the text. If the outcome of interest was not "all-cause mortality" (or a composite outcome including all-cause mortality), the number of competing events was also considered. Articles were then classified as "consistent" if the numbers calculated matched the reported numbers at risk. If inconsistencies were noted between the LTFU information derived from the plot and given in the text or if LTFU information could be derived from the plot and no further information was provided in the text or the calculated number at risk was larger than the reported one, the articles were classified as "not consistent" (see Figure 2 for an example calculation).

All articles were assessed by either EV or MK. A subset of articles (those published in 2005) was assessed by both authors and no relevant discrepancies in the assessment were noted. Articles that were classified as "not consistent" and articles where classification was initially unclear were reassessed by a second reviewer (MK, EV, TK, or GS). Disagreement was resolved by consensus.

In order to evaluate the robustness and validity of study results, sensitivity analyses were performed for all study publications classified as "not consistent". In these publications we calculated a higher number of patients at risk than was reported in the Kaplan-Meier plot and which could not be explained by the reported LTFU. We aimed to assess the potential risk of bias caused by this discrepancy. For this purpose, we generated a 2 × 2 contingency table for time point t (one time point before minimum follow-up, as defined above) by calculating the number of events of interest up to this time and then performed a χ^2^-test. We generated a second contingency table where the difference between calculated and reported numbers at risk, minus the reported LTFU, was imputed (unreported LTFU). If no LTFU were reported their number was assumed to be zero and the total difference was imputed. We classified a treatment effect as "robust" if the effect estimate did not change direction and the corresponding p-value remained significant or not significant (α = 5%) after imputation. In the equal-case scenario, the unreported LTFU data were imputed as "event" in both groups. In the worst-case scenario, unreported LTFU data were imputed as "event" in the test group and "no event" in the control group (best-case scenario: vice versa).

## Results

**Figure 3:**
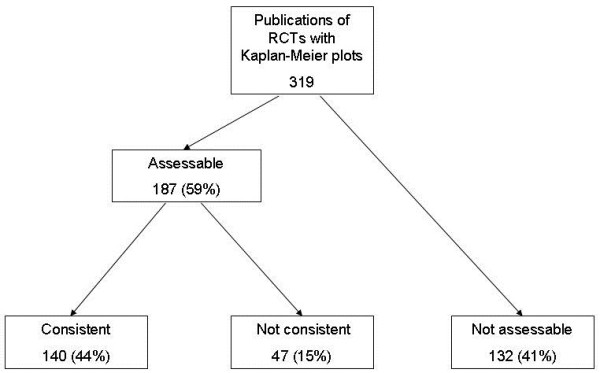
**Main results of the assessment of Kaplan-Meier plots in articles on randomised controlled trials (RCTs)**.

Of 734 articles on RCTs, 319 were eligible for inclusion (Figure 3). Of these 319 articles, 187 (59%) were classified as "assessable", as they included sufficient information for the assessment of LTFU; 132 articles (41%) were not assessable.

140 of 319 articles (44%) presented consistent LTFU information between the Kaplan-Meier plot and the text. 47 (15%) were classified as "not consistent", either because a higher rate of LTFU was derived from the plot than was presented in the text (18 of 319 articles; 6%) or the LTFU information could be derived from the plot but no further information was found in the text (29 of 319 articles; 9%).

These 47 articles were included in the sensitivity analyses. When an equal-case scenario was used as an imputation method, the results changed and hence were not robust in 21 (45%) of these studies (table 1). As expected this proportion was even higher in the best- and worst-case scenario (55% and 57% respectively; table 1).

**Table 1:** Change in study results* after imputation of censored data

		Imputation method		
**Original treatment effect**	**N**	**Equal case** **n (%)**	**Best case** **n (%)**	**Worst case** **n (%)**

Significant	24	8 (33)	7 (29)	9 (38)
Not significant	23	13 (57)	19 (83)	18 (78)

Total	47	21 (45)	26 (55)	27 (57)

* After imputation of censored data the effect estimate changed direction and the corresponding p-value changed from significant to not significant or vice versa (α = 5%).

The journals reporting the fewest and the most Kaplan-Meier plots were the BMJ (14 of 319; 4%) and the NEJM (138 of 319; 43%) respectively (table 2). The proportion of articles classified as "not assessable" varied from 19% in JAMA and 93% in the BMJ, the latter finding being due to the fact that, with one exception, plots presented in the BMJ did not report numbers at risk in the figure. In the remaining journals the proportion of articles classified as "consistent" ranged from 33% (NEJM) to 64% (JAMA).

**Table 2:** Results of the assessment of LTFU information stratified by journal

	BMJ	JAMA	Lancet	NEJM	Total
Articles (n)	14	70	97	138	319

Not assessable* (n (%))	13 (93)	13 (19)	32 (33)	74 (54)	132 (41)

Consistent** (n (%))	1 (7)	45 (64)	49 (51)	45 (33)	140 (44)
Not consistent (n (%))	0	12 (17)	16 (16)	19 (14)	47 (15)

* Loss to follow-up information cannot be derived from the Kaplan-Meier plot.

** The numbers derived from the Kaplan-Meier plot matched the reported numbers at risk.

## Discussion

In this survey of over 300 articles on RCTs published in four leading medical journals and using Kaplan-Meier plots, less than half of the studies presented assessable and consistent LTFU information. This poor reporting of items of survival analyses is in line with the results of previous research. Reviews of articles on cancer trials presenting survival analyses found that less than 10% of articles reported survival outcomes optimally [8], and only about half included any summary of length of follow-up [7]. Regarding the reporting of LTFU, only about a quarter of articles mentioned whether LTFU occurred or not and if LTFU information was given, only about half of the articles stated how they were treated in the analyses [7].

Another problem in papers using survival analyses is that they frequently do not account for competing risks [8]. In the case of competing risks the Aalen-Johansen estimator should be preferred to the Kaplan-Meier estimator [14]. When competing events are censored, the Kaplan-Meier curve cannot be interpreted as probabilities [15] and may produce inconsistent information on LTFU. It would therefore be interesting to investigate how many articles in major medical journals deal adequately with competing risks. However, the focus of this paper was only on the reporting quality of LTFU information.

A part of the eligible pool of articles was originally assessed by only one reviewer. However, articles where classification was initially unclear and articles classified as "not consistent" were always checked by a second reviewer; by minimising the number of wrong allocations to this category we thus consider our findings to be conservative. Contacting study authors might have been helpful in clarifying some of the inconsistencies found; however, as our focus was on the reporting quality of survival analyses in published articles, no contact was made. Nevertheless, within the framework of our regular work we were able to verify inconsistencies in three publications included in the survey. In two cases we had access to the full clinical study report. In the third case, the author informed us that the inconsistency was due to a mistake in the editorial processing of the Kaplan-Meier plot.

Several recommendations for improving the numerical and graphical presentation of survival analyses have been provided in the literature [5,6,16]. Additional methods to support data presentation have also been proposed: for example, Royston et al. [17] developed an approach to illustrate the distribution of observed and censored survival times; Clark et al. [18] suggested a completeness index to quantify the effect of LTFU, which could be helpful in identifying possible bias caused by unequal follow-up. Another approach to increase the quality of survival data could be the improvement of study design to increase protocol adherence, e.g. inclusion of run-in periods to identify non-compliant patients [19]. The reasons for LTFU or missing data should always be provided, as depending on the reason (e.g. worsening of disease), different imputation methods may be required [20].

The CONSORT explanation and elaboration document extended its recommendations on the reporting of follow-up time in 2010, and in addition to stating the median duration of follow-up, now also recommends stating the minimum and maximum duration [21]. We suggest that CONSORT should also recommend reporting the numbers at risk and competing events, as well as provide some advice on the numerical and graphical presentation of survival analyses to help authors present these data appropriately. As already suggested in relation to CONSORT [22], we also propose that in their instructions for authors, journals should be more explicit as to the extent to which authors should adhere to specific recommendations.

The LOST to follow-up Information in Trials (LOST-IT) study is currently being conducted with the primary objective of assessing the potential impact of LTFU on the estimates of treatment effect in RCTs with binary outcomes [23]. This study is expected to have important implications for trialists and users of the medical literature and further proposals to minimise LTFU are anticipated as a consequence of LOST-IT [23].

## Conclusions

Our survey shows that less than half of the articles on RCTs using Kaplan-Meier plots provide assessable and consistent LTFU information, thus questioning the validity of the results and conclusions of many studies presenting survival analyses. Authors should improve the presentation of both Kaplan-Meier plots and information on LTFU, and reviewers of study publications and journal editors should critically appraise the validity of the information provided.

## Competing interests

The authors declare that they have no competing interests.

## Authors' contributions

Study concept and design: EV, TK. Acquisition of data: EV, MK, TK. Analysis and interpretation of data: EV, MK, GS, RB, TK. Drafting of the manuscript: EV. Critical revision of the manuscript for important intellectual content: RB, TK, GS. Administrative, technical, or material support: EV, MK. Study supervision: TK.

All authors read and approved the final manuscript.

## Pre-publication history

The pre-publication history for this paper can be accessed here:

http://www.biomedcentral.com/1471-2288/11/130/prepub

## Supplementary Material

Additional file 1 **Search strategy**. This file contains the search strategy of our search for randomized controlled trials in PubMed. Click here for file
