# Supplementary material for: Reporting of loss to follow-up information in randomised controlled trials with time-to-event outcomes: a literature survey
Source: BMC Med Res Methodol. 2011 Sep 21;11:130. doi: 10.1186/1471-2288-11-130 (PMC3189898; doi:10.1186/1471-2288-11-130)
Supplement: Additional file 1 — Search strategy. This file contains the search strategy of our search for randomized controlled trials in PubMed. [file 1471-2288-11-130-S1.DOCX]

**Search strategy**

Search strategy:

1. (randomized controlled trial[pt] OR randomized controlled trials[mh] OR random allocation[mh] OR double-blind method[mh] OR single-blind method[mh]) OR ((single[tiab] OR double[tiab]) AND (mask*[tiab] OR blind*[tiab])) OR ((randomis*[tiab] OR randomiz*[tiab]) AND controlled[tiab] AND (trial[tiab] OR study[tiab]))
2. "Br Med J"[Journal:__jrid1912] OR "N Engl J Med"[Journal:__jrid5985] OR "JAMA"[Journal:__jrid5346] OR "Lancet"[Journal:__jrid5470]

Limits: only items with abstracts, Publication Date from 2003/01/01 to 2005/12/31
